# Perspective: Might Maternal Dietary Monosodium Glutamate (MSG) Consumption Impact Pre- and Peri-Implantation Embryos and Their Subsequent Development?

**DOI:** 10.3390/ijerph192013611

**Published:** 2022-10-20

**Authors:** Lon J. Van Winkle

**Affiliations:** 1 Department of Biochemistry, Midwestern University, Downers Grove, IL 60515, USA; lvanwinkle@rvu.edu; 2 Department of Medical Humanities, Rocky Vista University, 8401 S. Chambers Road, Parker, CO 80112, USA

**Keywords:** blastocyst, brain, embryo, environmental insult, epigenetics, glutamate signaling, lysine metabolism, metabolic syndrome, MSG, uterus

## Abstract

MSG alters metabolism, especially in the brain, when administered to experimental animals via gavage or similar means. Such administration is, however, not applicable to humans. More recently, though, MSG was shown to have these effects even when added to the food of mammals. Moreover, the levels of MSG in food needed to cause these metabolic changes are the same as those needed for optimum flavor enhancement. Near physiological concentrations of glutamate also cause mammalian blastocysts to develop with fewer cells, especially in their inner cell masses, when these embryos are cultured with this amino acid. We propose that consumption of MSG in food may overwhelm the otherwise well-regulated glutamate signaling needed for optimal development by pre- and peri-implantation mammalian embryos. In addition to immediate changes in cellular proliferation and differentiation as embryos develop, MSG ingestion during early pregnancy might result in undesirable conditions, including metabolic syndrome, in adults. Since these conditions are often the result of epigenetic changes, they could become transgenerational. In light of these possibilities, we suggest several studies to test the merit of our hypothesis.

## 1. Introduction

MSG is the monosodium salt of the nonessential amino acid, glutamic acid [1]. At neutral pH, glutamic acid is present in living organisms, primarily as the five-carbon anionic amino acid, glutamate. It forms an important amino acid residue in proteins, and it is metabolized to the five-carbon intermediate of the citric acid cycle, alpha-ketoglutarate (2-oxoglutarate). Most importantly to the present discussion, glutamate serves as an essential signaling molecule in many tissues and organs, including the brain [1].

MSG administration has been shown in numerous studies to adversely affect several mammalian body organs, including kidney, liver, ovaries, testis, and especially the brain, e.g., [1,2]. In most studies, however, MSG was given systemically by gavage or oral bolus. Thus, the relevance of these studies to humans can be questioned, in part, because humans consume MSG in food [2]. In this perspective paper, we present evidence that MSG can cause beneficial, as well as harmful, changes when added to food, and we cite studies showing that MSG can apparently reach embryos/fetuses in the uterus when administered to mice on days 10–20 of pregnancy. The latter is important—our premise is that MSG, consumed as a flavor enhancer in food, may reach pre- and peri-implantation embryos in the uterus and overwhelm the carefully regulated glutamate signaling needed for normal blastocyst implantation and continued development.

## 2. MSG Ingestion Appears to Alter Brain Glutamate Signaling and Whole-Body Metabolism: A Delicate Balance?

When added to food, MSG has beneficial, as well as deleterious, effects on animal brain morphology, brain function, disposition, behavior, and metabolic status. For example, in one study, mice consuming food-added MSG in a standard diet for eight weeks had greater locomotion, less grooming behavior, and lower anxiety than mice fed the same diet without MSG [2]. Somewhat surprisingly, this MSG consumption led to greater food intake but a lower gain in body weight. MSG had similar effects on mice consuming a high fat diet, except that they also ate less food and had better memories than animals consuming the same diet without MSG [2]. In addition, MSG seemed to protect mice from the detrimental effects of a high-fat diet on cerebral cortex morphology.

In another study, MSG was added to standard rat food or a “western diet” at a level needed for optimal flavor enhancement in humans [3]. After 14 weeks consuming these diets, MSG improved the memories of rats fed the Western diet, while it impaired memory in rats eating chow [3]. To our knowledge, the effects of food-added MSG on human brain function does not appear to have been studied. Interestingly, however, a low glutamate diet for one month improved cognitive function in veterans with Gulf War illness [4].

## 3. MSG Apparently Reaches Embryos/Fetuses in the Uterus after Ingestion by Their Mothers

The administration of MSG to dams from day 10 to 20 of pregnancy produced pups that grew more slowly than control pups [5]. This effect can likely be attributed to a direct effect of MSG on pups rather than their mothers, because alterations in the pups resemble changes also observed in mammals treated directly with MSG. (See Section 2 above). In addition to slower weight gain, pups from mice treated with MSG exhibited anxiety- and depression-like behaviors [5]. These effects on brain development may have occurred, in part, because MSG treatment also led to a two-fold increase in the expression of the K^+^/Cl^−^ co-transporter (KCC2) in pup brains. Alterations in KCC2 expression appear to cause neurological disorders that affect behavior, possibly by causing inaccurate neuronal migration during brain development [5,6].

## 4. Unwanted Effects of Glutamate on Preimplantation Development

Earlier in development, direct exposure of embryos to two mM glutamate had no observable effect on mouse blastocysts [7]. However, 5.0 and 10.0 mM glutamate decreased the numbers of cells in both the inner cell mass (ICM) and trophectoderm (TE) when present in culture with embryos for 24 h [7]. These blastocysts also contained more dead cells than blastocysts cultured without this amino acid.

Mouse blastocysts were also found to express a number of glutamate receptor proteins [7]. The metabotropic glutamate receptor, GRM5, was expressed in both TE and ICM cells, as were the kainate receptors, GRIK3, GRIK4, and GRIK5, and the AMPA receptors, GRIA3 and 4. Consistent with the findings for glutamate and its receptors, AMPA and kainate each reduced the number of TE cells in cultured blastocysts, while another inotropic agonist, NMDA, had no effect on embryo cell number [7]. At a lower AMPA concentration, ICM cells were affected more than TE cells, as was the case for the GRM5 agonist, (S)-3,5-DHPG. The apparently adverse effects of glutamate on blastocysts were largely prevented by a combination of the GRM5 and AMPA/kainate receptor antagonists, CNQX and MPEP [7].

## 5. Possible Importance of Glutamate Receptors to Normal Pre- and Peri-Implantation Embryo Development

While glutamate may be toxic to blastocysts via glutamate receptors, ICM cells likely maintain their own pluripotency and proliferation through the regulated activation of some of these receptors [8]. Because they are derived from the ICM, mouse embryonic stem (mES) cells serve as a model for their progenitors [9]. Mouse ES cells produce glutamate to activate GRM5 receptors in vitro to sustain their own self renewal [8,10], so ICM cells likely activate these receptors in a similar manner for the same purpose in vivo [9]. Glutamate activation of GRM5 in mES cells maintains their pluripotency and proliferation by fostering the expression of the transcription factors, Oct-4 and Nanog, and through interactions with leukemia inhibitory factors to maintain higher c-Myc expression [8,10].

Similarly, human ES (hES) cells likely express GRM3 and GRM5, since the cells contain mRNA encoding the receptors [11]. We suggest that human, mouse, and probably other mammalian ICM cells convert lysine to glutamate and carefully regulate glutamate release from the cells for the autocrine maintenance of their pluripotency and proliferation [9,11].

## 6. Compartmentalized Conversion of Lysine to Glutamate Likely Supports Embryonic Stem Cell Pluripotency and Proliferation

Unlike ES cells, the TE helps to regulate the environment of ICM cells through interactions between these cell types in blastocysts. In this emerging model, both the TE and ICM itself control ICM surroundings. Thus, data concerning ES cell metabolism must be combined with knowledge of TE function to understand how ICM cells are regulated in vivo.

In this regard, lysine is not transported well by any known membrane transport system in the TE apical membrane [9,12]. However, it is likely needed for glutamate production in the ICM [11]. Consequently, lysine is probably generated through pinocytic uptake of protein from uterine fluid and its subsequent hydrolysis in trophoblast cells [9,11]. Lysine must then be released to the ICM via the TE basolateral membrane. To our knowledge, lysine uptake into ICM and ES cells has not as yet been characterized.

Nevertheless, hES cells release glutamate to the culture medium and remove lysine from it [11], so the cells must take up lysine via at least one transporter. Moreover, subsequent conversion of lysine to glutamate is conceivable because less glutamate is released from hES cells than the amount of lysine they consume [11]. However, why else do we think a pool of glutamate, specialized for GRM5 signaling, is produced from lysine in hES cells, why do we think this glutamate specifically maintains hES cell pluripotency and proliferation, and are there clinical consequences of altered lysine metabolism owing to, say, undernutrition (see below)?

To answer the first of these questions, human ES cells express an unexpectedly high levels of RNA encoding alpha-aminoadipic semialdehyde synthase (AASS) [11], and conversion of lysine to glutamate is regulated by this enzyme in human and mouse brain [13,14,15,16,17]. Thus, ES and their progenitor cells in the ICM also probably control glutamate generation in the same way [11]. As suggested above, this pool of glutamate is released selectively to maintain pluripotent and proliferating stem cells in the ICM [9,11]. However, other sources of metabolically produced glutamate include glutamine. Indeed, glutamate is generated in brain in the glutamate–glutamine cycle, but brain health requires more than this cycle to produce glutamate [13,14,15,16,17]. Moreover, increased glutamine uptake via system N in mouse ICM cells helps maintain diapause in blastocysts [18,19], but cell proliferation is very slow in such blastocysts. Glutamine uptake by ICM cells must be dramatically downregulated to foster their proliferation and break diapause [18,19]. In addition, ES and probably their progenitor cells express relatively low glutaminase activity [11]. Hence, the production of glutamate from glutamine is likely slow in ICM cells and seems unlikely to help maintain their pluripotency and proliferation through glutamate signaling. Instead, glutamate produced from glutamine in ES cells is further metabolized to α-ketoglutarate, which, along with epigenetic mechanisms, helps to maintain ES and probably their ICM progenitor cells [20,21].

For these reasons, the glutamate ICM cells produced from lysine appear to be used selectively for autocrine GRM5 signaling by these cells [11]. Perhaps mitochondria in ICM cells extrude the glutamate they produce from lysine, and then the cells preferentially release this glutamate for autocrine signaling. Alternatively, the TE could conceivably provide glutamate to the ICM directly. However, as mouse blastocysts develop, their glutamate content decreases [22,23]. Hence, the TE may be working to protect ICM cells from potentially harmful excess glutamate [9]. Excess glutamate reaching the ICM via the TE might overwhelm the carefully regulated glutamate signaling in ICM cells. (See below.) In support of the notion that abnormal maternal nutrition can significantly alter this glutamate signaling, F_2_ blastocysts from small for gestational age F_1_ mice have greatly increased glutamate production and lysine consumption, compared to blastocysts from normal sized mothers [24]. Since these cultured blastocysts formed outgrowths in vitro, ICM cells likely exhibited this abnormal amino acid metabolism. Importantly, lysine consumption greatly exceeded glutamate production, so the proposed lysine conversion to glutamate is feasible.

In summary, the maintenance of pluripotent stem cells in the ICM seems to depend on their conversion of lysine to glutamate [11]. The lysine deprivation of hES cells nearly blocks their proliferation, although such is not the case for mES cells [25,26]. However, similar to hES cells, the bovine ICM consumes lysine and produces glutamate [27]. Moreover, the bovine TE produces lysine [27], and likely supplies it to the ICM for glutamate synthesis, as predicted by us for the human and mouse TE [9]. If any of these processes are disturbed, the ICM may be modified epigenetically, resulting in metabolic syndrome and related disorders in adults [11,28]. Such epigenetic modification likely occurred to produce the F_2_ blastocysts from the small for gestational age mice discussed above, so the modifications seem to be transgenerational.

## 7. How Might Exogenous Glutamate Overwhelm This Regulated Way of Providing Glutamate Signaling for ICM Maintenance?

As discussed above, 5.0 and 10.0 mM glutamate decreased the numbers of cells in both the ICM and TE when present in culture with embryos for 24 h while two mM glutamate had no observable effect on mouse blastocysts [7]. Similarly, at a relatively low concentration of the GRIA3 and 4 agonist, AMPA, ICM cells were affected more than TE cells, as was the case for the GRM5 agonist, (S)-3,5-DHPG. A combination of the GRM5 and AMPA/kainate receptor antagonists, CNQX and MPEP, largely prevented the apparently adverse effects of glutamate on blastocysts [7].

Possibly for these reasons, preimplantation mouse embryos appear to be protected from undesirable changes in their glutamate content. The presence of 1.0 mM glutamate in their culture medium did not increase the glutamate content of 4–8-cell embryos cultured from the two-cell stage in vitro [23]. Similarly, blastocysts grown from the two-cell stage in vitro in the presence of five amino acids abundant in oviductal fluid, did not have a lower glutamate content when 1.0 mM glutamate was left out of the medium. In contrast, the blastocysts had significantly less of each of the other four amino acids when they were deleted, one at a time, from the culture medium [23]. These results are somewhat surprising because the blastocyst TE contains a high-affinity, Na^+^-dependent, and concentrative glutamate transport system in its apical membrane [29], and the level of glutamate in blastocysts is greatly increased when 1.0 mM glutamate is present in the culture medium in the absence of other amino acids [23].

However, what if the glutamate concentration significantly exceeds 1.0 mM? We saw above that 5.0 mM glutamate causes a decrease in blastocyst cell number, and cells in the ICM were particularly susceptible to this effect. Moreover, the normal concentrations of glutamate in bovine and mouse uterine fluids approach 5.0 mM [30,31]. This concentration decreases significantly in mouse uterine fluid, from about 4.7 to 3.5 mM, as blastocysts approach implantation [31]. Similarly, the glutamate content of blastocysts decreases significantly as implantation nears [23]. Thus, we see that glutamate levels in blastocysts and the fluid that surrounds them appear to be carefully regulated during the peri-implantation period.

However, can MSG ingestion overwhelm this regulation? Experiments with the low-birth-weight female mice discussed above hints that alterations in amino acid availability to F_1_ mice can disrupt the regulation of glutamate production from lysine in their F_2_ blastocysts in a transgenerational manner. However, the overproduction of glutamate from lysine in the latter case was associated with increased cell number in the blastocysts [24], whereas greater exogenous glutamate in culture caused a decrease in cell numbers in both the TE and ICM [7]. These differences likely reflect both differences in the glutamate concentrations and the way higher glutamate was supplied. A higher supply of exogenous glutamate might occur when excess MSG is ingested by mothers during pre- and peri-implantation blastocyst development.

**Figure 1:**
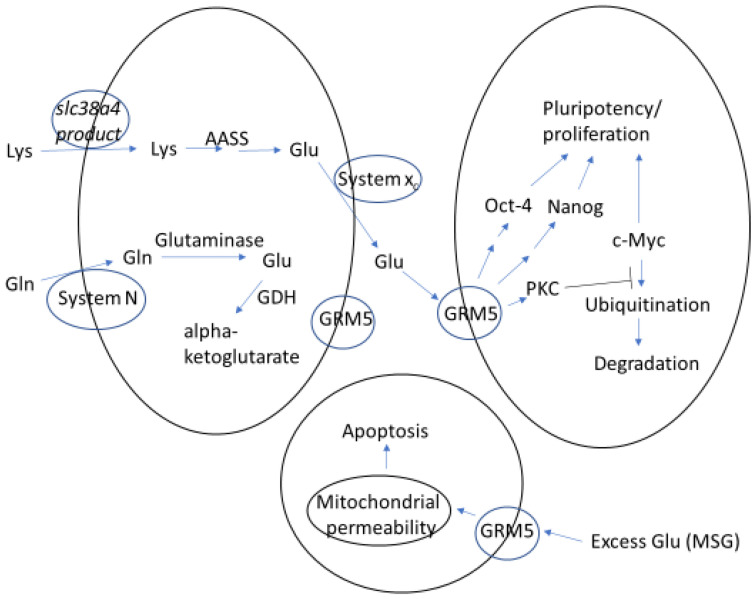
Proposed mechanism for carful regulation of glutamate (Glu) production and signaling in the ICM of blastocysts and how excess Glu may lead to reduced cell number in the ICM, in part, via apoptosis. For simplicity, only three cells are depicted, and processes that likely occur in all cells are shown in only one cell at a time. Plasma and mitochondrial membranes are illustrated by black ovals and amino acid transport systems and glutamate receptors by blue ovals in the plasma membranes. We propose that lysine (Lys) is taken up by a product of *slc38a4* (a Lys preferring system in other cells and the mRNA of which has been detected in blastocyst cDNA libraries) [22] and converted to Glu [11]. The resultant Glu is released from cells possibly via amino acid transport system x_c_ [22] and initiates signaling by metabotropic glutamate receptor 5 (GRM5) [8,10]. This signaling increases the levels of transcription factors, Oct-4 and Nanog, needed to maintain pluripotency and proliferation of the cells [8]. The signaling also raises the level of c-Myc by activating protein kinase C (PKC), which slows c-Myc degradation (see [10] for details of this process) and promotes pluripotency and proliferation. We also suggest that excess Glu (MSG) leads to signaling via GRM5 and other Glu receptors in the ICM, which causes a reduction in cell number [7], in part, by somehow activating mitochondrial pathways of apoptosis.

In this regard, the normal glutamate concentrations in uterine secretions are near harmful levels [30,31]. Thus, excess dietary glutamate intake as MSG could influence blastocyst development. For example, rats consuming a Western diet supplemented with an amount of MSG needed for optimal flavor enhancement, had 46% higher average blood serum (and plasma) glutamate concentrations than rats fed the same diet without MSG [3]. If the glutamate concentration increases by a similar amount in uterine fluid, then the normal average values of 4.7 to 3.5 mM during a 24 h period near the time of blastocyst implantation in mice [31] would increase to 6.8 and 5.1 mM, respectively. Additionally, the culture of blastocysts in medium containing 5.0 mM glutamate, significantly reduces blastocyst cell numbers, especially in the ICM [7]. Since MSG intoxication causes apoptosis in rat liver and kidney [32], we suggest that excess glutamate also causes reduction in ICM cell number, in part, via apoptosis in the blastocyst (Figure 1). The likely need for carefully regulated glutamate production and signaling in the blastocyst ICM are illustrated in Figure 1.

## 8. Need for Further Studies

MSG added to food at a level intended to promote optimal flavor enhancement alters mammalian body and brain metabolism and morphology [2,3]. Conversely, low MSG diets benefit persons with Gulf War illness [4]. Hence, it should be determined whether low MSG diets improve other neurological disorders. Similarly, MSG likely impairs neurological development in embryos/fetuses [5], but it needs to be determined whether this MSG reaches conceptuses at a great enough concentration to influence development. Additionally, it needs to be determined whether the administered MSG alters development through an effect on mothers carrying embryos/fetuses rather than on the embryo/fetuses themselves. Moreover, it should be determined whether MSG administration influences subsequent pregnancies of these mothers, including possible epigenetic changes, in the absence of further MSG consumption.

We also know that direct exposure of preimplantation blastocysts to glutamate alters their development, especially in their ICMs [7]. These alterations occur via the action of glutamate on its receptors. Moreover, alterations in the amino acid supply to early embryos, for example, owing to nutrient deprivation of F_1_ dams in utero, influences subsequent amino acid utilization and production by F_2_ pre- and peri-implantation blastocysts from the dams that produce the embryos [24]. For these reasons, it should be determined whether direct exposure of blastocysts to glutamate influences their metabolism, such as production and utilization of various amino acids. In addition, the fates of glutamate-treated blastocysts should be determined after transfer to surrogate mothers.

The metabotropic glutamate receptor, GRM5, is known to be particularly important for maintenance of ES and probably their progenitor cells in the ICM [8,10]. Hence, the need for subsequent glutamate signaling in early embryos should be determined as development proceeds. Experimental approaches could include use of glutamate receptor agonists and antagonists, as well as the detection of expression of glutamate receptors in various embryonal tissues.

Especially important to our premise is whether food-added MSG can produce levels of glutamate in oviductal and uterine secretions that are harmful to embryos. For example, 5.0 mM glutamate in vitro causes unwanted effects on blastocyst cell number [7], but, as discussed above, what impact, if any, does such treatment have on further development of these conceptuses and offspring derived from them? In addition, we need to learn whether food with added MSG raises the concentration of about 4.0 mM glutamate, normally found in uterine secretions [30,31], to the potentially harmful levels of 5.0 mM or more. Additionally, if food with added MSG has this effect, do glutamate levels of 5.0 mM or more adversely affect subsequent early embryo development in vitro and in utero? Again, to rule out an effect of MSG on mothers, embryo transfer to surrogate mothers should also be employed.

In particular, what is the relationship of this food-added MSG to the seemingly careful regulation of glutamate production from lysine in the ICM? Does MSG overwhelm this glutamate production or have no effect on it? Additionally, in mice in which the enzyme regulating this glutamate production, AASS, has been knocked out, is MSG consumption beneficial or harmful? Moreover, since this knock-out is not lethal [33,34], do knock-out offspring, nevertheless, have increased susceptibility to metabolic syndrome and related disorders as adults?

## 9. Conclusions

In summary, it has become clear that food-added MSG alters metabolism especially in the mammalian brain. Moreover, MSG causes these effects even at the best flavor -enhancing levels. Similarly, glutamate has unwanted effects on blastocysts cultured at near physiological concentrations of this amino acid. Our premise is that food-added MSG can overwhelm the carefully regulated glutamate signaling in pre- and peri-implantation embryos that is needed for their optimum development. Not only might MSG have immediate effects on tissue differentiation and growth in embryos, but it could also cause unwanted disorders, such as metabolic syndrome, later in life. Since epigenetic alterations might underly these changes in metabolism, they could also be transgenerational. For these reasons, we outline a number of studies above to test our hypotheses.

## Data Availability

Not applicable.
